# Systematic review of fluorescence-guided surgery in pituitary neuroendocrine tumours

**DOI:** 10.1530/EO-25-0037

**Published:** 2025-10-01

**Authors:** Christopher Dillon Ovenden, Nicholas Candy, James McNeil, Ryan Beerling Dolovac, Mendel Castle-Kirszbaum, Amer Helal, Sunita De Sousa, Alkis Psaltis

**Affiliations:** ^1^ The University of Adelaide Faculty of Health and Medical Sciences, Adelaide, Australia; ^2^ Surgery, The University of Adelaide Faculty of Health and Medical Sciences, Adelaide, Australia; ^3^ Nuclear Medicine, Royal Adelaide Hospital, Adelaide, Australia; ^4^ Endocrinology, Royal Adelaide Hospital, Adelaide, Australia; ^5^ The University of Western Australia Faculty of Medicine, Dentistry and Health Sciences, Perth, Australia; ^6^ Neurosurgery, Monash Health, Clayton, Australia; ^7^ Surgery, Monash University Faculty of Medicine, Nursing and Health Sciences, Melbourne, Australia; ^8^ Royal Adelaide Hospital, Adelaide, Australia; ^9^ Endocrine & Metabolic Unit, Royal Adelaide Hospital, Adelaide, Australia; ^10^ Department of Otolaryngology, Head and Neck Surgery, The Queen Elizabeth Hospital, Adelaide, Australia

**Keywords:** fluorescence, fluorescence-guided surgery, endoscopic, pituitary, pituitary neuroendocrine tumour, pituitary adenoma

## Abstract

**Objective:**

Fluorescence-guided surgery represents a potential novel technique to improve the extent of tumour resection, minimise resection of normal tissue, and optimise outcomes in pituitary neuroendocrine tumour surgery. We systematically reviewed the available evidence on this topic to define the current role for fluorescence-guided surgery in this setting.

**Design and methods:**

A systematic review was conducted in accordance with the PRISMA 2020 guidelines. MEDLINE, EMBASE, and the Cochrane Library were searched on 1st March 2025 for studies investigating the use of exogenous fluorophores or autofluorescence in pituitary surgery. Two authors independently screened studies and extracted data from included studies. Risk of bias assessment was performed using the Newcastle–Ottawa Scale.

**Results:**

From 1,604 studies identified, 22 studies involving 297 patients were included. Risk of bias was high in the vast majority of studies. Studies on six exogenous fluorophores were identified (indocyanine green, OTL38, bevacizumab-800CW, chlorin E6 photosensitiser, 5-aminolevulinic acid, and sodium fluorescein), with three studies assessing autofluorescence. Findings of included studies were variable, with small sample sizes and limited outcome reporting. 5-Aminolevulinic acid seems to have little use as a fluorophore in pituitary surgery. The clinical utility of the remaining fluorophores is not yet clearly defined. There are limited data on the utility of autofluorescence in pituitary surgery.

**Conclusion:**

At present, there is insufficient evidence to support the routine use of fluorescence-guided surgery in this setting, prompting the need for further research in this area.

## Introduction

Pituitary neuroendocrine tumours (PitNETs; also referred to as pituitary adenomas) are common intracranial tumours with a prevalence of 10–20% (1). Surgery is first-line treatment in the setting of large non-functioning PitNETs (NFP) with neural compromise, and in some types of functional PitNETs (FP) (2, 3, 4). Preservation of the normal pituitary gland is critical to minimise post-operative hypopituitarism, and identification of tumour is vital to potentiate maximal resection and avoid residual disease. This can be challenging as tissue discrimination under standard endoscopic surgical conditions can be difficult. Although the goal of surgery differs in NFP compared to FP, difficulty in achieving complete tumour resection, with pituitary gland preservation, is a common challenge. In the setting of NFP, 40–50% patients have been shown to exhibit residual disease following surgery, and this has not significantly improved over the last few decades (5). 30% patients fail to achieve long-term remission following surgery for growth hormone (GH)-producing tumours, and the number is 20% for ACTH-producing tumours (6, 7).

Techniques that facilitate differentiation of tumour, normal gland, and surrounding anatomical structures could facilitate safer surgery, with greater rates of gross total resection (GTR). Fluorescence-guided surgery has been used in other forms of intracranial surgery with promising results (8).

Autofluorescence is the process where biological tissue is excited by light of a certain wavelength, subsequently emitting light of a longer wavelength. The structural and molecular make-up of a tissue dictates the resultant wavelength on autofluorescence emission spectra. Analysis of these spectra can be used to differentiate between certain tissues. Utilisation of the intrinsic autofluorescence properties of other endocrine organs, such as the parathyroid glands and the adrenals, has been documented, but little is known regarding the autofluorescence properties of PitNETs or the normal pituitary gland (9, 10). Recently, there has been a proliferation of studies assessing the role of certain fluorescent agents in pituitary surgery, with a review by Vergeer *et al.* 2022 reviewing the efficacy of several fluorescent agents in the setting of endoscopic PitNET surgery, with mixed results (11). Since that review, multiple novel agents have been assessed in this area, and no previous review has examined the utility of autofluorescence in PitNET surgery.

This study aims to systematically review the use of autofluorescence and fluorescent agents to date in surgery on PitNETs.

## Materials and methods

The protocol for this systematic review was prospectively registered on 24th February 2025 on PROSPERO (ID CRD420250656537). Conduct of this review adhered to the stipulations of the Preferred Reporting Items for Systematic Reviews and Meta-Analyses 2020 guidelines (12).

### Eligibility criteria and search strategy

Inclusion criteria were structured according to the PICO framework (13). The population was patients with operatively managed PitNETs, the intervention was the use of fluorescent dyes or autofluorescence, the comparator was patients who did not have these techniques used, and the outcomes were measures of the safety and efficacy of the surgery. Randomised controlled trials, prospective and retrospective cohort studies, and case reports were included. Review articles were excluded. Only English-language articles were included. Studies investigating the role of fluorescence-guided surgery in non-PitNET skull base surgery were excluded.

MEDLINE, EMBASE, and the Cochrane Library were searched from database inception to 1st March 2025. The search strategy reflected the PICO format detailed above and was aimed at finding any study assessing the utility of fluorescent dyes or autofluorescence in the setting of PitNET surgery. The full search strategies used for each database can be found in full in Supplementary Appendix 1 (see section on Supplementary materials given at the end of the article).

### Selection process and data extraction

Two authors (CDO and RBD) utilised the web and mobile app Rayyan (Qatar Computing Research Institute, Qatar) to independently screen titles and abstracts (14). Following this, full texts were screened independently to determine final inclusion. At each stage, disagreements were resolved by consensus. Each included study was then independently reviewed and predetermined data were extracted. Information collected from each study was year of publication, number of patients, type of fluorophore investigated, timing of administration of fluorophore, dose of fluorophore, fluorescence characteristics of tumour, gland, and surrounding structures if available, and extent of resection achieved. It was presumed that the data obtained would be heterogeneous in nature, so no plans for statistical testing or meta-analysis were made. Instead, data were collated to be presented in tabular format and summarised in a narrative manner.

### Risk of bias and quality assessment

Risk of bias was analysed in included studies through use of the Newcastle–Ottawa Scale (NOS) tool, which allows for assessment of non-randomised observational studies, which the majority of the included studies were presumed to be (15). This scale involves scoring studies on the basis of three main characteristics: selection of studies, comparability of the cohort to a control, and outcome measurements (15). Scales are assigned a score from zero to nine based on how they align with the aforementioned criteria. Conversion of this score to a grading of poor, fair, or good was also performed as per an additional previous publication (16).

## Results

### Study selection

**Figure 1:**
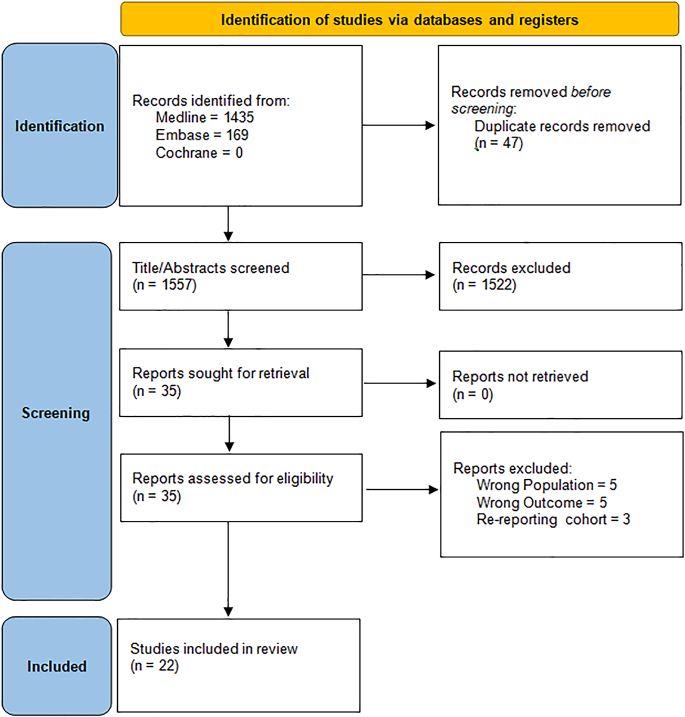
PRISMA flow diagram.

Our search terms returned a total of 1,604 studies, from which 35 studies were identified for full-text review. Of these, 22 were identified as eligible for inclusion, with Fig. 1 summarising the study selection process.

### Study characteristics

**Figure 2:**
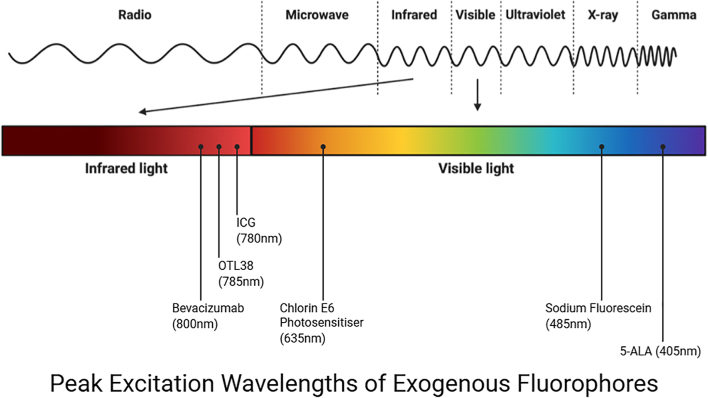
Peak excitation wavelengths of exogenous fluorophores.

Overall, 297 patients were involved in these studies, with six different exogenous fluorophores used in 284 patients, in addition to three papers assessing pituitary autofluorescence in 13 patients. Supplementary Table 1 details the study characteristics and risk-of-bias assessments for each included study. Note that the study by Cho and colleagues has been listed twice in the table, as it assessed a cohort that had indocyanine green and a separate cohort that had OTL38 (17). Mean NOS score was 4.8 (±1.0) out of nine, with two studies rated as good, one as fair, and the remainder rated as poor. The data were qualitatively heterogeneous and are summarised below in a narrative format based on the specific fluorophore assessed. Figure 2 summarises the peak excitation wavelengths of the exogenous fluorophores investigated.

### Indocyanine green dye

Indocyanine green (ICG) is a water-soluble dye that accumulates in regions of increased vascular permeability, is excited by light of a wavelength of 780–800 nm, and maximally emits light of a wavelength of 835 nm (18). ICG is the most commonly studied fluorophore in PitNET surgery, with 12 papers studying 167 patients found (17, 19, 20, 21, 22, 23, 24, 25, 26, 27, 28, 29). In the five studies that reported on the extent of resection, GTR was achieved in 57 of 74 (77%) patients (17, 19, 20, 21, 27). Although not an intra-study comparison, this rate is slightly higher than rates of GTR in PitNET surgery not utilising ICG in the general literature (5). Significant variability was seen in the dose and timing of ICG administration. Although most studies had ICG given at the time of, or shortly before, tumour resection, Cho and colleagues examined second-window ICG, where a larger dose was given 24 h before surgery (17, 25). Unless using the delayed-window technique, ICG fluorescence of tumour tended to occur quickly and rapidly wash out, with many cases requiring multiple doses (20).

The evidence is mixed with regard to timing of tumour versus normal gland fluorescence following ICG administration, with some studies reporting similar timing, some reporting earlier tumour fluorescence, and others reporting earlier gland fluorescence (20, 24, 26). The fluorescent characteristics of tumour were consistently noted as being similar to that of extravasated blood and surrounding dura (19, 23, 26). In contrast, normal gland or pituitary stalk was consistently shown to be more fluorescent than both tumour and other normal surrounding tissue, regardless of the timing of ICG administration (19, 22, 23, 25, 28). In papers that quantitatively assessed relative fluorescence between normal gland and tumour, normal glandular tissue was found to be 1.5–4.5 times more fluorescent than PitNET (19, 22, 28).

### OTL38

OTL38 is a folate analogue bound to ICG that is internalised after binding to folate receptor α (30). Three studies investigating OTL38 in PitNET surgery were identified, but these studies are all from the same institution, and on close analysis of the cohorts in each of these studies it is clear that data for some patients have been reported in multiple studies (17, 31, 32). Of these, the most comprehensive paper includes 23 patients who were administered OTL38, so this was included for further discussion in this review (17). Three hours before surgery, patients were given 0.025 mg/kg of OTL38, with intraoperative excitation occurring at 785 nm and recording at 800–835 nm. Normal glandular tissue did not fluoresce. None of the nine included FP demonstrated significant fluorescence. Of the fourteen NFP, nine (64%) demonstrated significant fluorescence that was distinguishable from surrounding tissue. These NFP were subsequently investigated for overexpression of folate receptor α, with perfect correlation of receptor overexpression and fluorescence seen. None of the nine FP assessed were investigated for overexpression of folate receptor α.

### Bevacizumab-800CW

Bevacizumab is a monoclonal antibody with competitive inhibition of vascular endothelial growth factor A and may be conjugated to the fluorophore IRDye 800CW, resulting in a molecule that fluoresces when exposed to light in the near-infrared spectrum (maximally at 750–800 nm) (33). A single-centre feasibility and dose-optimisation trial was recently published examining the utility of bevacizumab-800CW (BV800) in surgery for Knosp grade 3 or 4 PitNETs (34). In the dose-escalation phase of the study, four doses (0, 4.5, 10, and 25 mg) were trialled, each in a group of three patients. The techniques used to measure *in vivo* fluorescence were quantitative fluorescence molecular endoscopy (FME) and multi-diameter single fibre reflectance/single fibre fluorescence (MDSFR/SFF). The second technique to assess fluorescence is performed by the insertion of a spectroscopy fibre into the tissue in question. Following the initial phase, three additional patients were tested at the 10- and 25-mg doses, as these were the doses exhibiting significant differences in fluorescence between tumours and normal adjacent tissue. There were no adverse events attributed to BV800 in any of the study patients. Although significant differences in fluorescence were present between tumour and normal tissue when assessed with MDSFR/SFF, FME was unable to reliably differentiate tumour and normal tissue at any dose.

### Chlorin E6 photosensitiser

Chlorin E6 is a photosensitiser molecule that accumulates within tumour tissue as a result of the increased tumoural vascular permeability (35). One pilot study involving three patients was found investigating the use of this molecule as a fluorophore in PitNET surgery (36). Patients were administered a 1 mg/kg dose three hours before surgery and then had fluorescence assessed *in vivo* with an endoscope irradiating the field with light of wavelength 635 nm. Qualitatively, significant fluorescence of the PitNET was encountered in every patient. Quantitatively, fluorescence was 6–13 fold greater than that of the dura of the anterior sella. No quantitative comparison was made between the fluorescence of tumour and the fluorescence of normal gland.

### 5-Aminolevulinic acid

5-Aminolevulinic acid (5-ALA) is a molecule that certain cells will take up and convert into protoporphyrin IX, which, when exposed to light with a wavelength of 405 nm, will strongly emit at 635 nm (8). The first study that measured fluorescence in PitNETs administered 20 mg/kg 5-ALA 3 h before surgery in 30 patients. They reported a sensitivity of 95% and a specificity of 100% in 5-ALA fluorescence identifying PitNETs (37). This study did not use surgeon interpretation of fluorescence but rather laser probe spectrometry, requiring a small fibre to be inserted into the tumour. Two subsequent studies incorporating surgeon assessment of fluorescence by different groups found that in a combined 27 patients only one PitNET demonstrated significant detectable fluorescence (38, 39). 5-ALA dosages and timing of administration were similar between studies. In one instance, the normal pituitary gland demonstrated fluorescence (38).

### Sodium fluorescein

Sodium fluorescein is a fluorophore that, when excited by light of 475 nm, will strongly emit light of a wavelength of 500–550 nm, with accumulation seen in regions with compromise of the blood–brain barrier (40). The first report of the use of sodium fluorescein as an adjunct to PitNET surgery was in 2010 and included one patient as part of a larger cohort of patients undergoing open microscopic surgery for skull–base tumours (41). The light source was the standard white light provided by a microscope, and no specific filters were placed on the wavelengths of the recorded light. When digitally processed and analysed, the tumour was 40% more fluorescent in the wavelength sodium fluorescein would be expected to emit (the authors did not define this wavelength range in their paper) (41). Tumour samples were not examined *ex vivo* for fluorescence.

The only other study of the use of sodium fluorescein in PitNETs is a pilot study that prospectively assessed 15 patients (seven NFP, six GH producing, one ACTH producing, and one prolactin producing) receiving a dose of 8 mg/kg ten minutes before commencement of sellar dura opening (42). The YELLOW560 filter of the microscope was used to limit visualised wavelengths to 540–690 nm, and subsequent analysis of the 13 patients who had availability of the images for analysis showed statistically significant fluorescence of PitNET tissue compared to surrounding tissue. Although the sample size was too small to comment on statistical significance for this outcome, only 40% patients achieved complete resection of tumour (42). Again, patients did not have *ex vivo* assessment of fluorescence performed.

### Autofluorescence

Three studies were identified that assessed the autofluorescence of PitNETs *ex vivo* (43, 44, 45). In 2009, Saraswathy examined the autofluorescent properties of brain tumours *ex vivo* at light of wavelengths 320, 370, 410, and 470 nm, and the cohort included five PitNETs (45). Some degree of autofluorescence occurred in all cases and wavelengths, but the intensity of autofluorescence was consistently less than that of adjacent normal tissue. It was not clearly defined what the normal tissue being tested was, but the emission spectra mirrored that of the normal tissue tested in meningioma cases, and hence was presumably dura. Fürtjes and associates excited six PitNET samples with light of a wavelength of 790 nm (43). Although some autofluorescence occurred, this was half as intense as samples of normal cerebrum and 10% as intense as samples of dura. A separate study included two PitNETs while examining autofluorescence of different types of brain tumour irradiated with light of a wavelength of 488 nm *ex vivo* (44). One of the tumours had intense autofluorescence at this wavelength, the other did not. No information was provided on subtyping differences between the two PitNETs that could explain the difference in autofluorescence findings.

Although not a study with the direct aim of investigating autofluorescence, Schmidt and colleagues’ study on the use of bevacizumab as a fluorescent dye in pituitary surgery featured a control group of three patients who were not administered any of the study drug (34). Immunohistochemistry (IHC) results classified all three of these tumours as gonadotroph tumours. No tumour demonstrated significant autofluorescence when excited with light of a wavelength of 750 nm. Although a limited sample size, these results suggest significant autofluorescence does not occur when PitNETs are irradiated with light of this wavelength.

## Discussion

Optimisation of extent of resection while preserving normal pituitary gland function is a challenging but critical surgical goal in both FP and NFP (5, 6, 7). Fluorescence-guided surgery may represent a way to improve outcomes in this setting, but previous reviews have failed to define a fluorophore with clear evidence of efficacy (11). Our review details the findings of multiple recent studies investigating novel fluorophores as well as work assessing autofluorescence in PitNET surgery. The significance of our findings for individual fluorophores is detailed below.

### Indocyanine green dye

The role of ICG is well established in vascular neurosurgery, and its success in this field has prompted multiple studies on its role in skull-base surgery (46, 47). The studies assessing ICG in PitNET surgery are invariably small and are of mixed quality, with most being pilot-style studies intended to guide future research. None of the included studies compared extent of resection and surgical outcome in ICG cohorts to control cohorts. The significantly higher fluorescence of normal gland compared to tumour does, however, suggest possible utility of ICG in delineating this boundary and potentially maximising preservation of normal gland or stalk (19, 22, 28). However, there is no conclusive evidence at this stage that use of ICG confers benefit in pituitary preservation and avoidance of post-operative hypopituitarism. Furthermore, the fact that tumour tissue fluoresces in a manner similar to blood and surrounding tissues (such as the dura of the cavernous sinus) means that this fluorophore is unlikely to be able to potentiate identification of residual tumour and allow more complete resections (19, 23, 26). In cases with significant cavernous sinus invasion requiring parasellar dissection, ICG may play a role in clearly delineating the boundaries of the cavernous internal carotid artery and guiding tumour resection (48). However, further work is required to investigate the role, if any, of ICG in PitNET surgery.

### OTL38

OTL38 was initially investigated in the setting of breast, ovarian, and lung cancer (49, 50). Although FP tend not to overexpress folate receptor α, this is quite commonly seen in NFP (51, 52). The limited studies performed on OTL38 in this setting have shown limited utility when operating on FPs, but variable utility in NFP (17). Tumoural fluorescence appears dependent on the presence of overexpression of folate receptor α. The preoperative identification of overexpression of folate receptors would be key in predicting which patients would benefit from OTL38, and there is some early evidence that (99m)Tc-folate SPECT/CT could provide this information (53). Although there does seem to be a subset of PitNET patients who may benefit from OTL38 as a fluorophore, evidence is currently limited, and it is unclear how to define the patients who will most likely benefit.

### Bevacizumab-800CW

VEGF overexpression is seen in 30–50% of operated PitNETs, with higher rates in prolactinomas, tumours with a higher proliferative index, and pituitary carcinomas (54, 55, 56). Lloyd and colleagues highlighted that, though VEGF expression was in some cases higher in PitNET compared to normal gland, in other cases the opposite was true (56). This very likely will limit the possibility of BV800 being used as a fluorophore to distinguish between normal gland and tumour. With regard to differentiation of tumour from surrounding structures such as normal tissue, though the MDSFR/SFF technique Schmidt and colleagues used was able to differentiate fluorescence of tumour and surrounding structures, this is not necessarily a technique that can easily be replicated in a routine manner intraoperatively (34). The fact that FME was not able to reliably delineate tumour from normal surrounding tissue limits the utility of this technique, with the authors hypothesising a possible confounding effect of blood obscuring the field of interest (34). Clinical and radiological methods to predict tumours overexpressing VEGF may help in the future to define a population likely to benefit from use of this fluorophore intraoperatively.

### Chlorin E6 photosensitiser

Chlorin E6 is a photosensitising agent that has a tolerable safety profile in darkness (35). There are some preliminary studies indicating it may be useful as a fluorophore in patients undergoing resection of high-grade gliomas (57, 58). Although Kozlikina’s recent study on its use in three PitNET operations is promising, caution must be exercised when considering this small sample size and lack of *ex vivo* confirmation of fluorescence (36). The authors comment on a possible role of Chlorin E6 facilitating photodynamic therapy of residual PitNET, but this is purely theoretical at this stage. Larger cohort studies and robust trials are required to further elucidate the role for Chlorin E6 photosensitiser in this setting.

### 5-Aminolevulinic acid

The utility of 5-ALA is contingent on its accumulation in regions of compromised blood–brain barrier, then subsequent cellular uptake and conversion to protoporphyrin IX. Use of this agent has been shown in randomised controlled trials to increase extent of resection of high-grade glioma and improve progression-free survival, with an optimum dose of 20 mg/kg (8, 59). Although initial work showed promising results with regard to 5-ALA in PitNET surgery, this was likely secondary to recording results with a laser diode physically advanced to the region of the tumour (37). Subsequent studies using a more pragmatic approach of surgeon ability to differentiate tumour from surrounding structures have shown that 5-ALA is not a reliable fluorophore in this setting (38, 39). Indeed, in rare cases normal pituitary gland will fluoresce under these conditions, potentially complicating attempts to discriminate between normal gland and tumour (38). A possible explanation for this may be inferred from the findings of Nemes and colleagues, who demonstrated *in vitro* that, though PitNET cells take up 5-ALA, these cells have an efflux mechanism preventing accumulation to high intracellular concentrations (60). At this stage, the potential for 5-ALA to be an efficacious fluorophore in PitNET surgery appears limited.

### Sodium fluorescein

Sodium fluorescein is a white-light fluorophore that has been investigated extensively in the setting of both high-grade glioma and brain metastasis (61, 62). The extracellular accumulation is dependent on blood–brain barrier disruption. The literature assessing the utility of this fluorophore in PitNET surgery is sparse. Although the literature available does suggest there is a quantitative difference in the degree of fluorescence of tumour tissue compared to surrounding structures, there is limited evidence to suggest these differences can be subjectively determined intraoperatively (41, 42). Furthermore, there is no data to suggest that use of this agent improves outcome, with one study utilising this technique only achieving a GTR rate of 40% (42). Although this study was not designed or powered to answer this question, the results should engender caution about adopting the use of sodium fluorescein in PitNET surgery at this stage.

### Autofluorescence

Tissue autofluorescence depends on the concentration and characteristics of the endogenous fluorophores within tissue, with the extracellular matrix usually being the primary contributor (63). Certain endocrine organs, such as the adrenal gland, and parathyroid gland are known to be significantly autofluorescent when exposed to light in the near-infrared spectrum, though the responsible source for this is not clearly defined (9, 10, 64). Work by Ricciuti and colleagues has demonstrated the significant *ex vivo* autofluorescent properties of human pituitary gland (65). When stimulated by light with a wavelength of 488 nm, the gland emitted light most avidly in the 555–575 nm wavelength range. Limited information on the autofluorescence of normal pituitary gland at light of different wavelengths is currently available, particularly *in vivo*.

Current information on the autofluorescence of PitNETs is limited to small case series that have mainly assessed autofluorescence *ex vivo* (43, 44, 45). A restricted number of wavelengths has been examined, and at present the impact of PitNET subtype (i.e. functioning vs non-functioning) on autofluorescence has not been comprehensively assessed. The aforementioned *ex vivo* studies have consistently demonstrated that autofluorescence of tumour tissue tends to be less than surrounding normal tissue. This has been hypothesised to be secondary to high amounts of collagen in fibrous surrounding tissue such as dura mater (43). Another possible explanation is that endogenous fluorophores such as lipofuscin accumulate with increasing cellular age, and tumour cells have had inadequate duration to accumulate these substances compared to normal tissue (43). Of interest is the fact that *in vivo* studies of autofluorescence by Schmidt and colleagues did not show a significant difference between tumour and normal adjacent tissue, acknowledging that their measurements were confounded to an extent by haemorrhage obscuring the region of interest (34). Further work is needed to define the autofluorescent properties of different types of PitNETs at different light wavelengths both *in vivo* and *ex vivo*.

### Limitations

The studies investigating fluorescence-guided PitNET surgery have been limited by small-sized, single-institution series, significant methodological issues, and a lack of clearly defined outcome measures. At this stage, there is insufficient evidence to advocate for fluorescence-guided surgery in pituitary surgery, let alone any particular fluorophore. In order to delineate a place for any of the currently examined fluorophores, larger, more robust studies need to be performed that clearly demonstrate an outcome benefit. The majority of previously assessed fluorophores have been adopted from use in other clinical settings.

### Future directions

Future work focusing on developing PitNET-specific fluorophores from a first-principles approach could provide a more effective agent, but the optimal target is not currently clear (66). Further characterisation of the autofluorescence properties of normal pituitary gland and adenoma would potentially allow discrimination of normal and abnormal tissue without the use of a specific exogenous fluorophore. Modern endoscopy systems have the capacity to integrate fluorescence systems, which can then overlay the fluorescence and white-light image (67). These systems could potentially be leveraged in the future to allow autofluorescence- and fluorophore-guided surgery.

## Conclusion

Fluorescence-guided surgery is, in principle, a technique that could help improve resection rates and allow preservation of normal pituitary tissue. Although various fluorophores have been investigated in the setting of PitNET surgery, none of them have clearly established efficacy. Studies assessing this topic are largely limited to pilot studies demonstrating ‘proof of concept’, have a high risk of bias, and tend towards low methodological quality. There is insufficient evidence to support routine use of fluorophores in PitNET surgery at this point in time. Higher-quality studies of known fluorophores or identification of novel, targeted fluorophores may expand the role for fluorescence-guided surgery in this setting. Further characterisation of the autofluorescence properties of PitNETs and normal pituitary gland may also offer novel means of improving the efficacy and safety of PitNET surgery.

## Supplementary materials



## Declaration of interest

The authors declare that there is no conflict of interest that could be perceived as prejudicing the impartiality of the work reported.

## Funding

This work did not receive any specific grant from any funding agency in the public, commercial, or not-for-profit sector. The PhD of author CDO is supported by scholarships from the University of Adelaide, the Royal Australian College of Surgeons, and the Neurosurgical Research Foundation.

## Author contribution statement

CDO helped conceive the study, designed the paper, collected data, extracted data, analysed data, and wrote the manuscript. NC helped conceive the paper, helped design the paper, and contributed to critical review of the manuscript. JM analysed data and contributed to review of the manuscript. RBD helped with data collection, extraction, and analysis. AH helped with design of the study, data analysis, and critical review of the manuscript. MCK helped with design of the study, data collection, extraction, analysis, and critical review of the manuscript. SDS designed and conceptualised the study, analysed data and contributed to critical review and the manuscript. AP designed and conceptualised the study, analysed data and contributed to critical review and the manuscript.

## References

[1] Ezzat S, Asa SL, Couldwell WT, et al. The prevalence of pituitary adenomas: a systematic review. Cancer 2004 101 613–619. (10.1002/cncr.20412) 15274075

[2] Bray DP, Mannam S, Rindler RS, et al. Surgery for acromegaly: indications and goals. Front Endocrinol 2022 13 924589. (10.3389/fendo.2022.924589) PMC9386525 35992136

[3] Fleseriu M, Auchus R, Bancos I, et al. Consensus on diagnosis and management of Cushing’s disease: a guideline update. Lancet Diabetes Endocrinol 2021 9 847–875. (10.1016/s2213-8587(21)00235-7) 34687601 PMC8743006

[4] Muskens IS, Zamanipoor Najafabadi AH, Briceno V, et al. Visual outcomes after endoscopic endonasal pituitary adenoma resection: a systematic review and meta-analysis. Pituitary 2017 20 539–552. (10.1007/s11102-017-0815-9) 28643208 PMC5606952

[5] Roelfsema F, Biermasz NR & Pereira AM. Clinical factors involved in the recurrence of pituitary adenomas after surgical remission: a structured review and meta-analysis. Pituitary 2012 15 71–83. (10.1007/s11102-011-0347-7) 21918830 PMC3296023

[6] Schöfl C, Franz H, Grussendorf M, et al. Long-term outcome in patients with acromegaly: analysis of 1344 patients from the German acromegaly register. Eur J Endocrinol 2013 168 39–47. (10.1530/eje-12-0602) 23087126

[7] Broersen LHA, Biermasz NR, van Furth WR, et al. Endoscopic vs. microscopic transsphenoidal surgery for Cushing’s disease: a systematic review and meta-analysis. Pituitary 2018 21 524–534. (10.1007/s11102-018-0893-3) 29767319 PMC6132967

[8] Stummer W, Pichlmeier U, Meinel T, et al. Fluorescence-guided surgery with 5-aminolevulinic acid for resection of malignant glioma: a randomised controlled multicentre phase III trial. Lancet Oncol 2006 7 392–401. (10.1016/s1470-2045(06)70665-9) 16648043

[9] Di Marco AN & Palazzo FF. Near-infrared autofluorescence in thyroid and parathyroid surgery. Gland Surg 2020 9 (Supplement 2) S136–S146. (10.21037/gs.2020.01.04) 32175254 PMC7044090

[10] Rajan N, Scoville SD, Zhang T, et al. Adrenal near-infrared autofluorescence. J Endocr Soc 2022 6 bvac126. (10.1210/jendso/bvac126) 36111274 PMC9469928

[11] Vergeer RA, Theunissen REP, van Elk T, et al. Fluorescence-guided detection of pituitary neuroendocrine tumor (PitNET) tissue during endoscopic transsphenoidal surgery available agents, their potential, and technical aspects. Rev Endocr Metab Disord 2022 23 647–657. (10.1007/s11154-022-09718-9) 35344185 PMC9156450

[12] Page MJ, McKenzie JE, Bossuyt PM, et al. The PRISMA 2020 statement: an updated guideline for reporting systematic reviews. BMJ 2021 372 n71. (10.1136/bmj.n71) 33782057 PMC8005924

[13] Miller SA & Forrest JL. Enhancing your practice through evidence-based decision making: PICO, learning how to ask good questions. J Evid Base Dent Pract 2001 1 136–141. (10.1067/med.2001.118720)

[14] Ouzzani M, Hammady H, Fedorowicz Z, et al. Rayyan-a web and mobile app for systematic reviews. Syst Rev 2016 5 210. (10.1186/s13643-016-0384-4) 27919275 PMC5139140

[15] Wells GA, Shea B, O’Connell D, et al. The Newcastle–Ottawa scale (NOS) for assessing the quality of nonrandomised studies in meta-analyses. Ottawa, Ontario, Canada: University of Ottawa, 2000. (https://www.ohri.ca/programs/clinical_epidemiology/oxford.asp)

[16] Robinson CH, Albury C, McCartney D, et al. The relationship between duration and quality of sleep and upper respiratory tract infections: a systematic review. Fam Pract 2021 38 802–810. (10.1093/fampra/cmab033) 33997896 PMC8656143

[17] Cho SS, Jeon J, Buch L, et al. Intraoperative near-infrared imaging with receptor-specific versus passive delivery of fluorescent agents in pituitary adenomas. J Neurosurg 2019 131 1974–1984. (10.3171/2018.7.jns181642) 30554181 PMC10985533

[18] Teng CW, Huang V, Arguelles GR, et al. Applications of indocyanine green in brain tumor surgery: review of clinical evidence and emerging technologies. Neurosurg Focus 2021 50 E4. (10.3171/2020.10.focus20782) 33386005

[19] Muto J, Mine Y, Nishiyama Y, et al. Intraoperative real-time near-infrared image-guided endoscopic endonasal surgery for pituitary tumors. World Neurosurg 2023 175 e218–e229. (10.1016/j.wneu.2023.03.055) 36924890

[20] Inoue A, Kohno S, Ohnishi T, et al. Tricks and traps of ICG endoscopy for effectively applying endoscopic transsphenoidal surgery to pituitary adenoma. Neurosurg Rev 2021 44 2133–2143. (10.1007/s10143-020-01382-4) 32889658

[21] Lee MH & Lee TK. Application of fusion-fluorescence imaging using indocyanine green in endoscopic endonasal surgery. J Clin Neurosci 2022 98 45–52. (10.1016/j.jocn.2022.01.023) 35131724

[22] Shahein M, Prevedello DM, Beaumont TL, et al. The role of indocyanine green fluorescence in endoscopic endonasal skull base surgery and its imaging correlations. J Neurosurg 2021 135 923–933. (10.3171/2020.6.jns192775) 33186906

[23] Litvack ZN, Zada G & Laws ER Jr. Indocyanine green fluorescence endoscopy for visual differentiation of pituitary tumor from surrounding structures. J Neurosurg 2012 116 935–941. (10.3171/2012.1.jns11601) 22360574

[24] Hide T, Yano S, Shinojima N, et al. Usefulness of the indocyanine green fluorescence endoscope in endonasal transsphenoidal surgery. J Neurosurg 2015 122 1185–1192. (10.3171/2014.9.jns14599) 25723307

[25] Cho SS, Buch VP, Teng CW, et al. Near-infrared fluorescence with second-window indocyanine green as an adjunct to localize the pituitary stalk during skull base surgery. World Neurosurg 2020 136 326. (10.1016/j.wneu.2020.01.135) 31996340

[26] Amano K, Aihara Y, Tsuzuki S, et al. Application of indocyanine green fluorescence endoscopic system in transsphenoidal surgery for pituitary tumors. Acta Neurochir 2019 161 695–706. (10.1007/s00701-018-03778-0) 30762125

[27] Berardinelli J, Solari D, di Maria D, et al. Case report of indocyanine green endoscopy for intrasellar pituitary adenoma resection. World Neurosurg 2024 183 14. (10.1016/j.wneu.2023.12.008) 38070734

[28] Verstegen MJT, Tummers Q, Schutte PJ, et al. Intraoperative identification of a normal pituitary gland and an adenoma using near-infrared fluorescence imaging and low-dose indocyanine green. Oper Neurosurg 2016 12 260–268. (10.1227/neu.0000000000001328) 29506113

[29] Sandow N, Klene W, Elbelt U, et al. Intraoperative indocyanine green videoangiography for identification of pituitary adenomas using a microscopic transsphenoidal approach. Pituitary 2015 18 613–620. (10.1007/s11102-014-0620-7) 25492407

[30] Ledermann JA, Canevari S & Thigpen T. Targeting the folate receptor: diagnostic and therapeutic approaches to personalize cancer treatments. Ann Oncol 2015 26 2034–2043. (10.1093/annonc/mdv250) 26063635

[31] Lee JYK, Cho SS, Zeh R, et al. Folate receptor overexpression can be visualized in real time during pituitary adenoma endoscopic transsphenoidal surgery with near-infrared imaging. J Neurosurg 2018 129 390–403. (10.3171/2017.2.jns163191) 28841122 PMC10980838

[32] Cho SS, Zeh R, Pierce JT, et al. Folate receptor near-infrared optical imaging provides sensitive and specific intraoperative visualization of nonfunctional pituitary adenomas. Oper Neurosurg 2019 16 59–70. (10.1093/ons/opy034) 29635300 PMC7189272

[33] Ter Weele EJ, Terwisscha van Scheltinga AG, Linssen MD, et al. Development, preclinical safety, formulation, and stability of clinical grade bevacizumab-800CW, a new near infrared fluorescent imaging agent for first in human use. Eur J Pharm Biopharm 2016 104 226–234. (10.1016/j.ejpb.2016.05.008) 27179587

[34] Schmidt I, Vergeer RA, Postma MR, et al. Fluorescence detection of pituitary neuroendocrine tumour during endoscopic transsphenoidal surgery using bevacizumab-800CW: a non-randomised, non-blinded, single centre feasibility and dose finding trial [DEPARTURE trial]. Eur J Nucl Med Mol Imaging 2025 52 660–668. (10.1007/s00259-024-06947-9) 39390132 PMC11732902

[35] Hak A, Ali MS, Sankaranarayanan SA, et al. Chlorin e6: a promising photosensitizer in photo-based cancer nanomedicine. ACS Appl Bio Mater 2023 6 349–364. (10.1021/acsabm.2c00891) 36700563

[36] Kozlikina EI, Efendiev KT, Grigoriev AY, et al. A pilot study of fluorescence-guided resection of pituitary adenomas with chlorin e6 photosensitizer. Bioengineering 2022 9 52. (10.3390/bioengineering9020052) 35200407 PMC8869665

[37] Eljamel MS, Leese G & Moseley H. Intraoperative optical identification of pituitary adenomas. J Neuro Oncol 2009 92 417–421. (10.1007/s11060-009-9820-9) 19357967

[38] Micko A, Rapoport BI, Youngerman BE, et al. Limited utility of 5-ALA optical fluorescence in endoscopic endonasal skull base surgery: a multicenter retrospective study. J Neurosurg 2020 135 535–541. (10.3171/2020.5.jns201171) 33126212

[39] Marbacher S, Klinger E, Schwyzer L, et al. Use of fluorescence to guide resection or biopsy of primary brain tumors and brain metastases. Neurosurg Focus 2014 36 E10. (10.3171/2013.12.focus13464) 24484248

[40] Zhang N, Tian H, Huang D, et al. Sodium fluorescein-guided resection under the YELLOW 560 nm surgical microscope filter in malignant gliomas: our first 38 cases experience. Biomed Res Int 2017 2017 7865747. (10.1155/2017/7865747) 29124069 PMC5662847

[41] da Silva CE, da Silva JL & da Silva VD. Use of sodium fluorescein in skull base tumors. Surg Neurol Int 2010 1 70. (10.4103/2152-7806.72247) 21125008 PMC2980904

[42] Romano-Feinholz S, Alcocer-Barradas V, Benítez-Gasca A, et al. Hybrid fluorescein-guided surgery for pituitary adenoma resection: a pilot study. J Neurosurg 2019 132 1490–1498. (10.3171/2019.1.JNS181512) 30952130

[43] Fürtjes G, Reinecke D, von Spreckelsen N, et al. Intraoperative microscopic autofluorescence detection and characterization in brain tumors using stimulated Raman histology and two-photon fluorescence. Front Oncol 2023 13 1146031. (10.3389/fonc.2023.1146031) 37234975 PMC10207900

[44] Reichenbach M, Richter S, Galli R, et al. Clinical confocal laser endomicroscopy for imaging of autofluorescence signals of human brain tumors and non-tumor brain. J Cancer Res Clin Oncol 2024 151 19. (10.1007/s00432-024-06052-2) 39724474 PMC11671560

[45] Saraswathy A, Jayasree RS, Baiju KV, et al. Optimum wavelength for the differentiation of brain tumor tissue using autofluorescence spectroscopy. Photomed Laser Surg 2009 27 425–433. (10.1089/pho.2008.2316) 19025404

[46] de Oliveira JG, Beck J, Seifert V, et al. Assessment of flow in perforating arteries during intracranial aneurysm surgery using intraoperative near-infrared indocyanine green videoangiography. Neurosurgery 2008 62 (6 Supplement 3) 1300–1310. (10.1227/01.neu.0000333795.21468.d4) 18695550

[47] Imizu S, Kato Y, Sangli A, et al. Assessment of incomplete clipping of aneurysms intraoperatively by a near-infrared indocyanine green-video angiography (Niicg-Va) integrated microscope. Minim Invasive Neurosurg 2008 51 199–203. (10.1055/s-2008-1080916) 18683109

[48] de Notaris M, Sacco M, Corrivetti F, et al. Indocyanine green endoscopy for pituitary adenomas with parasellar extension: results from a preliminary case series. World Neurosurg 2022 166 e692–e702. (10.1016/j.wneu.2022.07.081) 35917924

[49] Toffoli G, Cernigoi C, Russo A, et al. Overexpression of folate binding protein in ovarian cancers. Int J Cancer 1997 74 193–198. (10.1002/(sici)1097-0215(19970422)74:2<193::aid-ijc10>3.0.co;2-f) 9133455

[50] Boogerd LS, Boonstra MC, Beck AJ, et al. Concordance of folate receptor-α expression between biopsy, primary tumor and metastasis in breast cancer and lung cancer patients. Oncotarget 2016 7 17442–17454. (10.18632/oncotarget.7856) 26943581 PMC4951224

[51] Liu X, Ma S, Yao Y, et al. Differential expression of folate receptor alpha in pituitary adenomas and its relationship to tumor behavior. Neurosurgery 2012 70 1274–1280 discussion 1280. (10.1227/neu.0b013e3182417e76) 22089756

[52] Evans CO, Reddy P, Brat DJ, et al. Differential expression of folate receptor in pituitary adenomas. Cancer Res 2003 63 4218–4224. 12874029

[53] Galt JR, Halkar RK, Evans CO, et al. In vivo assay of folate receptors in nonfunctional pituitary adenomas with 99mTc-folate SPECT/CT. J Nucl Med 2010 51 1716–1723. (10.2967/jnumed.108.061689) 20956474

[54] Iuchi T, Saeki N, Osato K, et al. Proliferation, vascular endothelial growth factor expression and cavernous sinus invasion in growth hormone secreting pituitary adenomas. Acta Neurochir 2000 142 1345–1351. (10.1007/s007010070003) 11214627

[55] Wang Y, Li J, Tohti M, et al. The expression profile of dopamine D2 receptor, MGMT and VEGF in different histological subtypes of pituitary adenomas: a study of 197 cases and indications for the medical therapy. J Exp Clin Cancer Res 2014 33 56. (10.1186/s13046-014-0056-y) 25027022 PMC4223393

[56] Lloyd RV, Scheithauer BW, Kuroki T, et al. Vascular endothelial growth factor (VEGF) expression in human pituitary adenomas and carcinomas. Endocr Pathol 1999 10 229–235. (10.1007/bf02738884) 12114703

[57] Akimoto J, Fukami S, Ichikawa M, et al. Intraoperative photodiagnosis for malignant glioma using photosensitizer talaporfin sodium. Front Surg 2019 6 12. (10.3389/fsurg.2019.00012) 30949484 PMC6438081

[58] Shimizu K, Nitta M, Komori T, et al. Intraoperative photodynamic diagnosis using talaporfin sodium simultaneously applied for photodynamic therapy against malignant glioma: a prospective clinical study. Front Neurol 2018 9 24. (10.3389/fneur.2018.00024) 29441040 PMC5797572

[59] Stummer W, Stepp H, Wiestler OD, et al. Randomized, prospective double-blinded study comparing 3 different doses of 5-aminolevulinic acid for fluorescence-guided resections of malignant gliomas. Neurosurgery 2017 81 230–239. (10.1093/neuros/nyx074) 28379547 PMC5808499

[60] Nemes A, Fortmann T, Poeschke S, et al. 5-ALA fluorescence in native pituitary adenoma cell lines: resection control and basis for photodynamic therapy (PDT)? PLoS One 2016 11 e0161364. (10.1371/journal.pone.0161364) 27583461 PMC5008746

[61] Schupper AJ, Rao M, Mohammadi N, et al. Fluorescence-guided surgery: a review on timing and use in brain tumor surgery. Front Neurol 2021 12 682151. (10.3389/fneur.2021.682151) 34220688 PMC8245059

[62] Ohadi MAD, Dashtkoohi M, Babaei MR, et al. Sodium fluorescein-guided resection of brain metastases: a needed approach or an option? A systematic review and meta-analysis. Acta Neurochir 2024 166 334. (10.1007/s00701-024-06223-7) 39133319

[63] Monici M. Cell and tissue autofluorescence research and diagnostic applications. Biotechnol Annu Rev 2005 11 227–256. (10.1016/s1387-2656(05)11007-2) 16216779

[64] Thomas G, McWade MA, Sanders ME, et al. Identifying the novel endogenous near-infrared fluorophore within parathyroid and other endocrine tissues. In Biomedical Optics 2016. Washington, DC, USA: Optica Publishing Group, 2016. PTu3A.5. (10.1364/BRAIN.2016.PTu3A.5)

[65] Ricciuti A, De Remigis A, Landek-Salgado MA, et al. Detection of pituitary antibodies by immunofluorescence: approach and results in patients with pituitary diseases. J Clin Endocrinol Metab 2014 99 1758–1766. (10.1210/jc.2014-1049) 24606106 PMC4010700

[66] Candy NG, Ramezanpour M, Bouras G, et al. Differential proteomic expression in non-functional pituitary neuroendocrine tumours and pituitary glands. Rhinology 2024 62 750–758. (10.4193/rhin24.216) 39140195

[67] Preziosi A, Cirelli C, Waterhouse D, et al. State of the art medical devices for fluorescence-guided surgery (FGS): technical review and future developments. Surg Endosc 2024 38 6227–6236. (10.1007/s00464-024-11236-5) 39294317 PMC11525393

