# Supplementary material for: Systematic review of fluorescence-guided surgery in pituitary neuroendocrine tumours
Source: Endocr Oncol. 2025 Oct 1;5(1):e250037. doi: 10.1530/EO-25-0037 (PMC12495904; doi:10.1530/EO-25-0037)
Supplement: Supplementary file 1 [file supplementary_materials.pdf]

Pubmed Search Terms

(pituitary adenoma[MeSH Terms] OR pituitary tumor[MeSH Terms] OR pituitary neoplasms[MeSH Terms] OR pituitar*[tiab])

AND (fluorescence[MeSH Terms] OR agents, fluorescent[MeSH Terms] OR near infrared spectrometric[MeSH Terms] OR infrared rays[MeSH Terms] OR fluorescen*[tiab])

Embase + Cochrane terms

((Fluorescence or autofluorescence) and (Pituitary Adenoma or Pituitary Neuroendocrine tumour or Pituitary) and Neurosurgery)

Type of fluorophore being studied	Study Number	Title	Authors	Year of Publication	Number of patients	Timing of administration	Dosage/Excitation wavelength	Fluorescence findings	Extent of Resection	NOS Selection Score	NOS Comparability Score	NOS Outcome score	NOS Overall Score	NOS Subjective grade
**Indocyanine Green Studies**	1	Intraoperative Real-Time Near-Infrared Image-Guided Endoscopic Endonasal Surgery for Pituitary Tumors.	Muto et al.	2023	25	When sellar dura exposed	12.5mg	SBR gland/tumour 4.5 ± 3.4 at 90 minutes. Consistently higher signal from gland	GTR = 22 STR =3	1	0	2	3	Poor
	2	Tricks and traps of ICG endoscopy for effectively applying endoscopic transsphenoidal surgery to pituitary adenoma.	Inoue et al.	2020	24	First dose just before opening sellar dura, second dose when tumour resection thought to be complete	Initial dose 12.5mg, second dose allowed if >30 minutes elapsed since first dose	Normal gland fluorescence at mean time 24.3 seconds post ICG, in tumour 53.5 seconds	GTR = 20, STR = 2, Partial Resection = 2	2	1	2	5	Fair
	3	Intraoperative near-infrared imaging with receptor-specific versus passive delivery of fluorescent agents in pituitary adenomas.	Cho et al.	2019	16	24 hours prior to surgery	5mg/kg	Mean SBR prior to dural opening 2.7 ± 0.63, mean following dural opening 4.1 ± 0.72	7 = GTR, 9 = STR	3	0	2	5	Poor
	4	Application of fusion-fluorescence imaging using indocyanine green in endoscopic endonasal surgery.	Lee et al.	2022	8	10-60 minutes prior to tumour debulking	12.5mg	6/8 cases had subjective ICG uptake	GTR =7, STR = 1	3	0	2	5	Poor
	5	Indocyanine green fluorescence endoscopy for visual differentiation of pituitary tumor from surrounding structures.	Litvack et al.	2012	9	Immediately prior to opening sellar dura	25mg	Tumour less fluorescent in 8/9 cases. Equivalent fluorescence in 1 case	NA	3	0	2	5	Poor
	6	Usefulness of the indocyanine green fluorescence endoscope in endonasal transsphenoidal surgery.	Hide et al.	2015	26	Once entering the sphenoid sinus	12.5mg	No significant difference in time taken for fluorescence to occur in normal gland vs pituitary adenoma	NA	3	0	2	5	Poor
	7	Near-Infrared Fluorescence with Second-Window Indocyanine Green as an Adjunct to Localize the Pituitary Stalk During Skull Base Surgery.	Cho et al.	2020	1	24 hours prior to surgery	2.5mg/kg	Following tumour resection, subjectively clear pituitary stalk under NIR	NA	NA	NA	NA	NA	NA

	8	Application of indocyanine green fluorescence endoscopic system in transsphenoidal surgery for pituitary tumors.	Amano et al.	2019	15	Following exposure of tumour and normal gland	6.25 or 12.5mg/kg	Tumour fluoresced at 6 minutes, no residual fluorescence at 9 minutes. Normal gland fluoresced later and longer	NA	3	0	1	4	Poor
	9	Intraoperative indocyanine green videoangiography for identification of pituitary adenomas using a microscopic transsphenoidal approach.	Sandow et al.	2015	22	Intraoperatively	25mg	Lower uptake of ICG compared to surrounding tissue	NA	3	0	2	5	Poor
	10	Case Report of Indocyanine Green Endoscopy for Intrasellar Pituitary Adenoma Resection.	Berardinelli et al.	2024	1	Prior to opening sellar dura	25mg	Commented was useful to differentiate tumour and gland	GTR	NA	NA	NA	NA	NA
	11	Intraoperative Identification of a Normal Pituitary Gland and an Adenoma Using Near-Infrared Fluorescence Imaging and Low-Dose Indocyanine Green.	Verstegen et al.	2016	10	Prior to opening sellar dura, subsequent doses done during tumour resection	5mg initial dose, allowed a further 2 doses	Ratio of fluorescence in pituitary gland to tumour 1.5 ± 0.2	NA	3	0	1	4	Poor
	12	The role of indocyanine green fluorescence in endoscopic endonasal skull base surgery and its imaging correlations.	Shahein et al.	2020	10	First dose just before opening sellar dura, second dose when tumour resection thought to be complete	12.5mg, allowed second dose of 12.5mg	Gland/Blood Fluorescence 0.722 ± 0.13, Tumour/Blood Fluorescence 0.538 ± 0.16	NA	4	1	2	7	Good
**Folate analog NIR dye OTL38**	13	Intraoperative near-infrared imaging with receptor-specific versus passive delivery of fluorescent agents in pituitary adenomas.	Cho et al.	2019	23	2-3 hours prior to surgery	0.025mg/kg	NFPA with FR overexpression mean SBR 3.2 ± 0.53 on tumour view. Other tumour subtypes SBR <2	GTR = 15, STR = 8	3	0	2	5	Poor

**5-ALA**	14	Use of fluorescence to guide resection or biopsy of primary brain tumors and brain metastases	Marbacher et al.	2014	12	3-5 hours prior to surgery	20mg/kg	Only 1 patient had subjective fluorescence	NA	3	0	2	5	Poor
	15	Intraoperative optical identification of pituitary adenomas.	Eljamel et al.	2009	30	3 hours prior to surgery	20mg/kg	Sensitivity 81% and Specificity 75% in detecting adenomas	NA	3	0	1	4	Poor
	16	Limited utility of 5-ALA optical fluorescence in endoscopic endonasal skull base surgery: a multicenter retrospective study.	Micko et al.	2020	15	2-4 hours prior to induction	20mg/kg	No adenomas demonstrated fluorescence, one normal gland had fluorescence	NA	3	0	2	5	Poor
**Bevacizumab-800CW**	17	Fluorescence detection of pituitary neuroendocrine tumour during endoscopic transsphenoidal surgery using bevacizumab-800CW: a non-randomised, non-blinded, single centre feasibility and dose finding trial [DEPARTURE trial].	Schmidt et al.	2024	18	3 days prior to surgery	0mg, 4.5mg, 10mg or 25mg	No significant fluorescence with FME, 10 and 25mg groups had significant fluorescence when measured with SFF	4 = GTR, 14 =STR	4	1	2	7	Good
**Autofluorescence**	18	Optimum wavelength for the differentiation of brain tumor tissue using autofluorescence spectroscopy.	Saraswathy et al.	2009	5	NA	Excitation wavelengths of 320, 370, 410 and 470nm all tested	Consistently lower autofluorescence of pituitary adenoma tissue in comparison to surrounding tissue	NA	3	0	3	6	Poor
	19	Clinical confocal laser endomicroscopy for imaging of autofluorescence signals of human brain tumors and non-tumor brain.	Reichenbach et al.	2024	2	NA	Excitation wavelength of 488nm	One case significant autofluorescence, the other not	NA	3	0	1	4	Poor

	20	Intraoperative microscopic autofluorescence detection and characterization in brain tumors using stimulated Raman histology and two-photon fluorescence.	Fürtjes et al.	2023	6	NA	Excitation wavelength of 790nm	Dura was 11 times more fluorescent than tumour. Normal cerebrum was around 2 times more fluorescent.	NA	3	0	2	5	Poor
**Chlorin e6 photosensitiser**	21	A Pilot Study of Fluorescence-Guided Resection of Pituitary Adenomas with Chlorin e6 Photosensitizer.	Kozlikina et al.	2022	3	3 hours prior to surgery	1mg/kg	Tumour 6 to 13 times more fluorescent than surrounding dura	NA	3	0	1	4	Poor
**Sodium Fluorescein**	22	Use of sodium fluorescein in skull base tumors.	da Silva et al.	2010	1	At time of tumour exposure	1g	40% increase in fluorescence of tumour following	NA	2	0	1	3	Poor
	23	Hybrid fluorescein-guided surgery for pituitary adenoma resection: a pilot study.	Romano-Feinholz et al.	2020	15	Following removal of rostrum	8mg/kg	On RGB scale (0-255), tumour tissue mean 146.2 ± 75.6, normal gland mean 29.6 ± 32.3, surrounding normal tissue mean 65.7 ± 42.2	GTR =6, STR =3, Partial =3	3	0	2	5	Poor

References

1. Muto J, Mine Y, Nishiyama Y, et al. Intraoperative Real-Time Near-Infrared Image-Guided Endoscopic Endonasal Surgery for Pituitary Tumors. World Neurosurg. 2023;175:e218-e229.

2. Inoue A, Kohno S, Ohnishi T, et al. Tricks and traps of ICG endoscopy for effectively applying endoscopic transsphenoidal surgery to pituitary adenoma. Neurosurg Rev. 2021;44(4):2133-2143.

3. Cho SS, Zeh R, Pierce JT, et al. Folate Receptor Near-Infrared Optical Imaging Provides Sensitive and Specific Intraoperative Visualization of Nonfunctional Pituitary Adenomas. Oper Neurosurg (Hagerstown). 2019;16(1):59-70.

4. Lee MH, Lee TK. Application of fusion-fluorescence imaging using indocyanine green in endoscopic endonasal surgery. J Clin Neurosci. 2022;98:45-52.

5. Litvack ZN, Zada G, Laws ER, Jr. Indocyanine green fluorescence endoscopy for visual differentiation of pituitary tumor from surrounding structures. J Neurosurg. 2012;116(5):935-941.

6. Hide T, Yano S, Shinojima N, Kuratsu J. Usefulness of the indocyanine green fluorescence endoscope in endonasal transsphenoidal surgery. J Neurosurg. 2015;122(5):1185-1192.

7. Cho SS, Buch VP, Teng CW, De Ravin E, Lee JYK. Near-Infrared Fluorescence with Second-Window Indocyanine Green as an Adjunct to Localize the Pituitary Stalk During Skull Base Surgery. World Neurosurg. 2020;136:326.

8. Amano K, Aihara Y, Tsuzuki S, Okada Y, Kawamata T. Application of indocyanine green fluorescence endoscopic system in transsphenoidal surgery for pituitary tumors. Acta Neurochir (Wien). 2019;161(4):695-706.

9. Sandow N, Klene W, Elbelt U, Strasburger CJ, Vajkoczy P. Intraoperative indocyanine green videoangiography for identification of pituitary adenomas using a microscopic transsphenoidal approach. Pituitary. 2015;18(5):613-620.

10. Berardinelli J, Solari D, di Maria D, Parbonetti G, Cavallo LM, de Notaris M. Case Report of Indocyanine Green Endoscopy for Intrasellar Pituitary Adenoma Resection. World Neurosurg. 2024;183:14.

11. Verstegen MJT, Tummers Q, Schutte PJ, et al. Intraoperative Identification of a Normal Pituitary Gland and an Adenoma Using Near-Infrared Fluorescence Imaging and Low-Dose Indocyanine Green. Oper Neurosurg (Hagerstown). 2016;12(3):260-268.

12. Shahein M, Prevedello DM, Beaumont TL, et al. The role of indocyanine green fluorescence in endoscopic endonasal skull base surgery and its imaging correlations. J Neurosurg. 2021;135(3):923-933.

13. Cho SS, Zeh R, Pierce JT, et al. Folate Receptor Near-Infrared Optical Imaging Provides Sensitive and Specific Intraoperative Visualization of Nonfunctional Pituitary Adenomas. Oper Neurosurg (Hagerstown). 2019;16(1):59-70.

14. Marbacher S, Klinger E, Schwyzer L, et al. Use of fluorescence to guide resection or biopsy of primary brain tumors and brain metastases. Neurosurg Focus. 2014;36(2):E10.

15. Eljamel MS, Leese G, Moseley H. Intraoperative optical identification of pituitary adenomas. J Neurooncol. 2009;92(3):417-421.

16. Micko A, Rapoport BI, Youngerman BE, et al. Limited utility of 5-ALA optical fluorescence in endoscopic endonasal skull base surgery: a multicenter retrospective study. J Neurosurg. 2020:1-7.

17. Schmidt I, Vergeer RA, Postma MR, et al. Fluorescence detection of pituitary neuroendocrine tumour during endoscopic transsphenoidal surgery using bevacizumab-800CW: a non-randomised, non-blinded, single centre feasibility and dose finding trial [DEPARTURE trial]. Eur J Nucl Med Mol Imaging. 2025;52(2):660-668.

18. Saraswathy A, Jayasree RS, Baiju KV, Gupta AK, Pillai VP. Optimum wavelength for the differentiation of brain tumor tissue using autofluorescence spectroscopy. Photomed Laser Surg. 2009;27(3):425-433.

19. Reichenbach M, Richter S, Galli R, et al. Clinical confocal laser endomicroscopy for imaging of autofluorescence signals of human brain tumors and non-tumor brain. J Cancer Res Clin Oncol. 2024;151(1):19.

20. Fürtjes G, Reinecke D, von Spreckelsen N, et al. Intraoperative microscopic autofluorescence detection and characterization in brain tumors using stimulated Raman histology and two-photon fluorescence. Front Oncol. 2023;13:1146031.

21. Kozlikina EI, Efendiev KT, Grigoriev AY, et al. A Pilot Study of Fluorescence-Guided Resection of Pituitary Adenomas with Chlorin e6 Photosensitizer. Bioengineering (Basel). 2022;9(2).

22. da Silva CE, da Silva JL, da Silva VD. Use of sodium fluorescein in skull base tumors. Surg Neurol Int. 2010;1:70.

23. Romano-Feinholz S, Alcocer-Barradas V, Benítez-Gasca A, Martínez-de la Maza E, Valencia-Ramos C, Gómez-Amador JL. Hybrid fluorescein-guided surgery for pituitary adenoma resection: a pilot study. J Neurosurg. 2019;132(5):1490-1498.
